# Approaches to Preoperative Assessment of Elderly Patients Undergoing Dental Surgical Interventions: A Systematic Review

**DOI:** 10.4317/jced.63672

**Published:** 2026-03-30

**Authors:** Aslan Ramazanovich Shurdumov, Artem Maksimovich Gusarov, Sergey Yuryevich Ivanov, Irina Vyacheslavovna Ivanova, Leonid Leonidovich Borozdkin, Shamsulvara Taymaskhanovich Kamilov, Alexey Mikhailovich Kuznetsov, Artem Dmitrievich Sviridenko

**Affiliations:** 1 Department of Maxillofacial Surgery I.M. Sechenov First Moscow State Medical University (Sechenov University), Moscow, Russia; 2 Department of Oral and Maxillofacial Surgery and Surgical Dentistry, Peoples’ Friendship University of Russia named after Patrice Lumumba (RUDN University), Moscow, Russia

## Abstract

**Background:**

Elderly patients represent a high-risk population for surgical dental interventions due to multimorbidity, frailty, and polypharmacy. Moreover, standard preoperative assessment protocols often fail to account for geriatric syndromes. Objective: To systematize and analyze contemporary approaches and tools for comprehensive preoperative assessment of elderly patients planning surgical dental procedures.

**Material and Methods:**

A systematic review was conducted in accordance with the PRISMA 2020 guidelines. The review protocol was registered in the international PROSPERO registry (ID: CRD420251147418). Searches were performed in PubMed/MEDLINE, Scopus, Web of Science, ScienceDirect, Google Scholar, CyberLeninka, and eLibrary.ru up to January 31, 2025. Original studies including patients aged 60 years focused on preoperative assessment prior to dento-maxillofacial surgery were included. Two reviewers independently performed study selection, data extraction, and risk-of-bias assessment using the ROBINS-I tool for observational studies.

**Results:**

Of 312 identified records, 12 studies were included in the analysis. The most frequently evaluated domains were nutritional status (MNA, MNA-SF; 5 studies), cognitive function (Mini-Cog, MMSE; 5 studies), frailty (Fried Criteria, CFS; 6 studies), and oral health (CPI, PI, OHIP-14; 8 studies). Meta-analysis was not performed due to high clinical and methodological heterogeneity. Overall evidence quality, assessed using the GRADE system for key outcomes, was low, confirming methodological limitations in the current scientific literature in geriatric dentistry.

**Conclusions:**

Despite the availability of numerous geriatric assessment tools, a standardized preoperative protocol for dental surgery is lacking. The findings emphasize the need for development and validation of a dental-specific assessment algorithm based on comprehensive geriatric assessment (CGA). Limitations: The conclusions should be interpreted with caution due to methodological limitations of the included studies, their heterogeneity, and inclusion of general maxillofacial surgery data, which reduced specificity for dental practice. Risk of publication bias was not assessed.

## Introduction

The aging population places special demands on dental surgery due to multimorbidity, reduced physiological reserves, and altered stress responses. Standard preoperative approaches often do not reflect the pathophysiological and functional characteristics of elderly patients (1 , 2). In Russia, the proportion of surgical interventions in patients over 60 years has significantly increased, and chronological age is no longer an absolute contraindication to surgery (3). Effective preoperative assessment plays a key role in outcome prediction, allowing consideration of multiple factors affecting surgical tolerance and complication risk (4). Modern risk scales frequently overlook biological age and functional status, necessitating more precise outcome prediction, including functional decline (5 - 7). Therefore, it is essential to identify risks associated with cognitive impairment, frailty, nutritional status, and polypharmacy to develop individualized optimization plans. Research Question and Objective. The primary question of this review was formulated according to PICO criteria: P (Population): Patients aged 60 years planning surgical dental interventions. I (Intervention/Exposure): Comprehensive preoperative assessment using geriatric scales and tools. C (Comparison): Standard preoperative evaluation or absence thereof. O (Outcomes): Postoperative complications (delirium, functional decline), surgical outcomes, applicability of assessment tools in dental practice. The aim of this systematic review is to systematize and analyze contemporary approaches and tools for comprehensive preoperative assessment of elderly patients prior to surgical dental interventions.

## Material and Methods

Study Design and Protocol Registration. The review was conducted following the PRISMA 2020 reporting guidelines. The review protocol was registered in the international prospective register of systematic reviews, PROSPERO (ID: CRD420251147418). Eligibility Criteria. Studies meeting the following criteria were included: Study types: Prospective and retrospective cohort studies, case-control studies. Participants: Patients aged 60 years planning surgical dental interventions (tooth extraction, dental implantation, bone grafting). Studies including general maxillofacial surgery patients were analyzed separately. Intervention/Assessment: Use of any validated tools or scales for comprehensive geriatric preoperative assessment. Comparison: Standard preoperative evaluation. Outcomes: Postoperative complications (delirium, infection), functional status, length of hospital stay, applicability in dental practice. Language: English, Russian. Exclusion Criteria: Systematic reviews, meta-analyses, case reports, editorials, animal studies, studies lacking data on assessment tools or outcomes. Information Sources and Search Strategy. Searches were conducted in PubMed/MEDLINE, Scopus, Web of Science, ScienceDirect, Google Scholar, CyberLeninka, and eLibrary.ru from January 1, 2017, to January 31, 2025. Example search string for PubMed: ("geriatric assessment"[MeSH Terms] OR "preoperative care"[MeSH Terms] OR "frailty"[MeSH Terms] OR "comprehensive geriatric assessment"[Title/Abstract]) AND ("oral surgical procedures"[MeSH Terms] OR "dental surgery"[Title/Abstract] OR "dentistry"[MeSH Terms]) AND ("aged"[MeSH Terms] OR "elderly"[Title/Abstract]) AND ("postoperative complications"[MeSH Terms] OR "delirium"[MeSH Terms]) AND (2017:2025[pdat]) Full search strings for key databases are provided in (Supplement 1) http://www.medicinaoral.com/medoralfree01/aop/jced_63672_s01. Study Selection, Data Extraction, and Risk of Bias Assessment. Study selection and data extraction were performed independently by two reviewers. Discrepancies were resolved through discussion or consultation with a third reviewer. The Rayyan tool was used for study screening. Data were extracted into a standardized table. Risk of bias in the included studies was assessed using the ROBINS-I tool for observational studies. Results for each domain are presented in (Supplement 2) http://www.medicinaoral.com/medoralfree01/aop/jced_63672_s02. Data extraction and management. Data extraction was performed independently by two review authors (Shurdumov A.R. and Gusarov A.M.) using a pre-tested standardized form created in Microsoft Excel. Any discrepancies that arose were resolved through discussion or by consulting a third author (Ivanov S.Yu.). The data extraction form was developed specifically for this review and included the following predefined items: Bibliographic data: first author, year of publication. Study characteristics: country, study design, objective. Participant characteristics: sample size, age, sex, type of surgical intervention. Assessment methods: all used preoperative assessment tools, grouped by domains (frailty, cognitive status, nutritional status, etc.). Outcomes: recorded primary and secondary outcomes. Key results: statistical measures of association (e.g., OR, RR with 95% CI). Authors' conclusions: main conclusions of the original study. The complete version of the form with extracted data for all included studies is presented in Supplementary File 1. Data Synthesis. Due to clinical and methodological heterogeneity among the included studies, meta-analysis was deemed inappropriate. Results are presented as a narrative synthesis with tabulated data. For key outcomes, the GRADE methodology was applied, and a "Summary of Findings" table was generated. Assessment of publication bias was not performed due to the inapplicability of standard methods (e.g., Egger's regression) to narrative synthesis. Planned effect measures. Nevertheless, the planned effect measures for each outcome were predefined as follows: For binary (dichotomous) outcomes (e.g., postoperative complications, delirium, tooth loss), the planned effect measures were the Odds Ratio (OR) or Risk Ratio (RR) with corresponding 95% confidence intervals (CI). For continuous outcomes (e.g., marginal bone loss, length of hospital stay, handgrip strength), the planned effect measures were the Mean Difference (MD) or Standardized Mean Difference (SMD) with 95% CI. If a sufficient amount of homogeneous data were available, we planned to pool these effect measures using a random-effects model.

## Results

**Figure 1:**
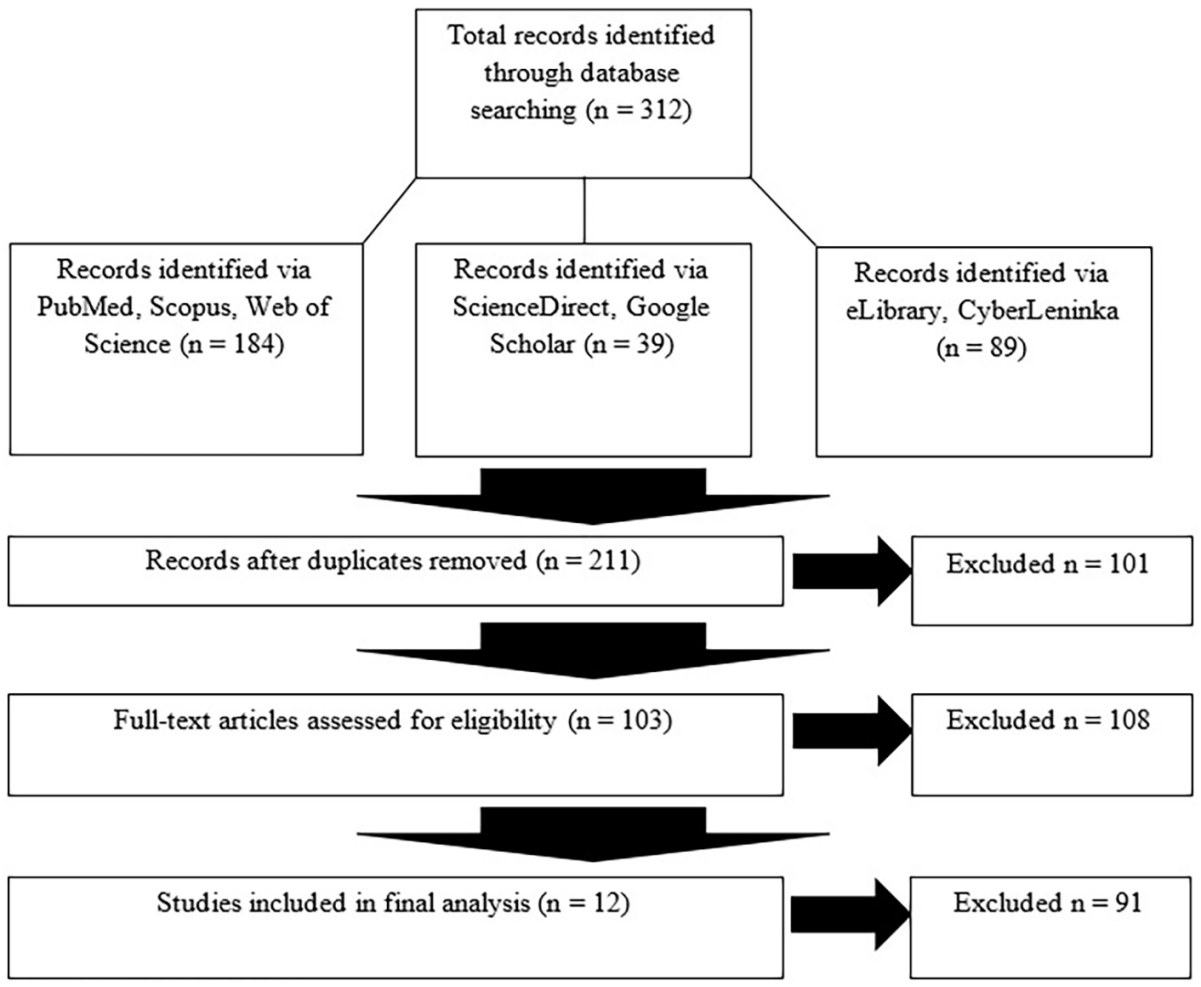
PRISMA 2020 flow diagram.

Search Results. Database searches identified 312 records. After removal of duplicates (n=101), 211 records were screened by title and abstract. Following exclusion of irrelevant records (n=108), 103 full-text articles were assessed for eligibility. After applying inclusion and exclusion criteria, 12 studies (8 - 19) were included in the final analysis. The study selection process is illustrated in Figure 1 (PRISMA 2020 flow diagram).

1

Characteristics of Included Studies. The included studies were published between 2017 and 2025. The total number of participants across all studies was 5,651. Detailed characteristics of the included studies are presented in Table 1.

Table 1

Risk of Bias Assessment (ROBINS-I). The majority of included studies (8 out of 12) demonstrated an overall moderate risk of bias, primarily due to potential confounding factors that were not accounted for and missing data. Three studies published in Russian journals were assessed as having a high risk of bias, mainly because of methodological limitations in study design, outcome measurement, and reporting. One study (11) was rated as having a low risk of bias. The high proportion of studies with moderate and high risk of bias was taken into account in the GRADE assessment of evidence quality, which was downgraded for all key outcomes (Supplement 2) http://www.medicinaoral.com/medoralfree01/aop/jced_63672_s02. Individual Study Results and Synthesis. The analysis revealed a wide range of tools used for preoperative assessment (Table 2).

Table 2

All tools were grouped into domains. The most frequently assessed areas were nutritional status (5 studies), cognitive function (5 studies), frailty and functional status (6 studies), and oral health (8 studies). Results from studies involving general maxillofacial surgery (12 , 13) were analyzed separately in the text to clearly distinguish dental-specific data. Grading of the quality of evidence (GRADE). For the key outcome (association of frailty/cognitive status with postoperative complications), the overall quality of evidence was rated as low due to risk of bias and heterogeneity among the included studies. Table 3 summarizes these findings.

Table 3

Risk of Bias: Serious limitations in the design of the primary studies. Indirectness: Some studies were conducted in the context of general maxillofacial surgery rather than specifically in dental practice, making the evidence less directly applicable to the target population. Inconsistency: High clinical and methodological heterogeneity (different study designs, interventions, sets of assessment tools, and outcomes). Imprecision: Meta-analysis was not performed, and effect estimates cannot be precisely determined. For some outcomes, the total number of patients was small. Inconsistency for Nutritional Status: The strength of association varied across different studies. Detailed quality of evidence profiles for each outcome, with full justifications for the GRADE decisions, are provided in (Supplement 3) http://www.medicinaoral.com/medoralfree01/aop/jced_63672_s03.

## Discussion

This systematic review demonstrates a wide range of tools used for preoperative assessment of elderly patients in dentistry and maxillofacial surgery. The findings confirm that effective assessment requires a multidisciplinary approach and extends beyond standard somatic examination. The most frequently assessed domains were nutritional status, cognitive function, frailty/functional status, and oral health. This aligns with contemporary understanding of geriatric syndromes as independent predictors of postoperative complications (20 , 21). A key observation is that frailty scales (e.g., Fried Criteria) and cognitive tests (e.g., Mini-Cog, CDT) show a strong association with postoperative complications, such as delirium and loss of functional independence (12 , 13 , 22 - 24). The primary limitation of this review and the analyzed literature is the significant heterogeneity in approaches and the absence of a unified consensus. Furthermore, inclusion of studies on general maxillofacial surgery, while broadening the range of assessment tools, reduced the specificity of conclusions for dento-maxillofacial surgery; this was addressed by analyzing these results separately. The findings should be interpreted with caution due to methodological limitations of the included studies (moderate risk of bias), high clinical and methodological heterogeneity, and inclusion of general maxillofacial surgery data. Meta-analysis was not performed. Assessment of publication bias was not conducted. Thus, the results underscore the importance of comprehensive geriatric assessment, while highlighting an urgent need for further high-quality research specifically focused on dental surgical practice, aimed at developing and validating a standardized, practice-oriented assessment algorithm.

## Figures and Tables

**Table 1:** Characteristics and Key Findings of Included Studies.

Researcher, Country, Year	Number ofParticipants	Age	Scale	Main Results
Penoni et al. (Brasil), 2018 [8]	134	65-80	BMD, FRAX	Periodontal care minimized the negative impact of low BMD on tooth-supporting tissues in the study population.
Luke Chow et al. (China), 2017 [9]	79	≥65	mMBL, GBL, PI	osteoporosis was not a contraindication for implant therapy, and reduced skeletal BMD was not associated with increased marginal bone loss around implants or other complications in an elderly population.
Seok Woo Hong et al. (South Korea), 2023 [10]	2322	≥65	FRAX, CPI, DXA, BMD, BMI, DMFT	Significant differences in BMI, existence of metabolic syndrome, number of present teeth, dental patterns, and CPI score were observed for both males and females, whereas the DMFT showed significant differences among groups only in the female elderly. There were significant differences in the aBMDs of the femoral neck among the three groups in males.
Shohei Nakatani et al. (Japan), 2023 [11]	331	≥65	Mini-Cog, MNA-SF	The prevalence of pre-operative undiagnosed cognitive impairment was 13%, and poor handgrip strength and worse oral hygiene were significantly associated factors.
Eman Alhammadi et al. (Germany), 2024 [12]	90	≥65	CDT, POD, CPS, DRAT, ACB, SARC-F, CFS, Katz Index, IADL, DEMMI, 4AT, NuDESC, CAM	A comprehensive approach that sets individualized goals for each patient is essential. Proper interventions in terms of the choice of surgical procedure and monitoring intraoperative factors such as operation duration should be applied. Findings identified multiple geriatric assessment instruments relevant to OMFS that can be easily assessed in the preoperative phase.
Rana Tuna Dogrul et al. (Turkey), 2020 [13]	108	≥65	ADL, IADL, MNA, MMSE, GDS, POSSUM, ASA, CCI, 4AT	In this study, CGA and frailty in preoperative period were found to be indicators for postoperative morbidity and delirium.
Rami K Aldwikat et al. (Australia), 2022 [14]	271	≥65	4AT,3D-CAM	The 3D-CAM and the 4AT are sensitive and specific screening tools that can be used to detect delirium in older people
Jing Ying Hu et al. (China) 2021 [15]	600	≥65	MDAS	The effects of dental anxiety on BP and HR in middle-aged and elderly patients with hypertension during local anaesthesia and tooth extraction were influenced by various confounding variables.
Bienvenu Bongue et al. (France), 2017 [16]	1224	≥65	aCGA, GFI, VES-13	The multivariate analyses showed that the VES-13 may predict the occurrence of disability, mortality and institutionalization.
Tsentroyev et al. (Russia), 2024 [17]	35	≥65	OHIP-14	Heartland computed tomography combined with minimally invasive treatment methods was shown to effectively improve oral health status in elderly patients. Overall patient perceptions indicated a positive effect of the surgical interventions, with most rating their post-treatment condition as improved.
Korenevich et al., (Russia), 2021 [18]	57	≥60	OHIP-14, MNA, Мини-ког	Alongside dental pathology (secondary complete and partial edentulism), patients experienced a decline in quality of life. Against this background, concomitant geriatric syndromes either developed or worsened.
Shakovets et al., (Russia), 2024 [19]	400	≥60	OHIP-14	The predicted risk of complications was confirmed at 94.17%; the model demonstrated a sensitivity of 94.36% and a specificity of 94.17%.

1

**Table 2:** The rating scale groups.

Scale group	Scale/index name	Short description/purpose
Cognitive scales	MMSE	Screening for cognitive impairment, dementia
	MoCA	Early diagnosis of mild cognitive impairment
	Mini-Cog	Rapid screening for dementia (memorization of words, clock test)
	SLUMS	Education-based cognitive screening
	Kokmen STMS	Mini-Mental State Examination
Fragility and Functional Consequence Scales	Fried Criteria	Frailty phenotype: weight loss, weakness, fatigue, etc.
	GFI (Groningen Frailty Indicator)	Comprehensive assessment of frailty (physical, cognitive, social spheres)
	Edmonton Frail Scale (EFS)	Multidimensional scale of frailty
	VES-13	Screening of vulnerable elderly patients
	Clinical Frailty Scale	Visual scale of clinical frailty
	FRAIL Scale	Rapid screening of frailty
Functional Activity Measures	Katz Index of ADL	Assessment of basic activities of daily living
	Barthel Index	Assessment of activities of daily living (0-100 points)
	Karnofsky Performance Score	Functional status (0-100%)
	Lawton-Brody IADL Scale	Assessment of instrumental activities of daily living
Nutritional Impact Scales	MNA-SF	Screening of nutritional deficiency
	MUST	Universal screening tool for malnutrition
	NRS 2002	Nutritional risk screening for hospitalized patients
	GNRI	Geriatric nutritional risk index
	PG-SGA	Subjective global assessment of nutrition
Bone and Fracture Risk Scales	BMD	Bone mineral density (DXA/DEXA)
	FRAX	Osteoporotic fracture risk calculator
	DXA/DEXA	BMD measurement method
Comorbidity and Polypharmacy	CCI	Comorbidity index
	CPS	Assessment polypharmacy
	ASA	Anesthetic risk classification
Dental and Periodontal Indices	CAL	Periodontium attachment loss
	CPI	Periodontium disease screening
	PDI	Comprehensive periodontal assessment
	GBI/PI	Bleeding and plaque indices
	OHAT	Comprehensive oral assessment
	CGOHAT	Comprehensive geriatric oral assessment
	Russell Periodontal Index	Classic periodontal index
	OHIP-14	Оценка влияния стоматологического здоровья
Surgical Risk Assessment	POSSUM (Physiological and Operative Severity Score)	Comprehensive scale of physiological and surgical complexity
	E-POSSUM	POSSUM version for the elderly
	ACS NSQIP Risk Calculator	Surgical risk calculator
Delirium Screening	4AT	Rapid delirium screening
	3D-CAM	Three-minute delirium diagnostic tool
	CAM	Confusion assessment
Anxiety Assessment	MDAS	Modified dental anxiety scale
	DAS	Dental anxiety scale
	DFS	Dental fear questionnaire
	IDAF-4C	Dental anxiety and fear index
Specialized Measures	G8	Geriatric screening tool
	Pfeiffer Test (SPMSQ)	Mini-mental status questionnaire
	TRST	Triady risk screening tool
	Share-FI	European frailty assessment tool
	Clock Drawing Test (CDT)	Clock drawing test for cognitive assessment

2

**Table 3:** Summary of Findings.

Outcome	Number of Studies (Participants); Design	Risk of Bias	Results and Effect (95% CI)	Evidence Quality (GRADE) with explanation
Association of frailty with postoperative complications (e.g., delirium, functional decline)	6 studies (n≈950); Observational cohorts	Serious¹	Presence of frailty (assessed using Fried Criteria, CFS) was associated with a 2–3-fold increased risk of postoperative complications (point estimate based on narrative synthesis). Meta-analysis not performed.	LOWMajor Limitations: Risk of bias: All included studies were observational cohorts with a moderate/high risk of bias, as assessed by the ROBINS-I tool.Indirectness: Some of the studies focused on general maxillofacial surgery rather than specific dental procedures.Heterogeneity: There was variability in the assessment tools used (e.g., Fried, CFS) and the reported outcomes.
Association of cognitive impairment with postoperative complications (e.g., delirium)	5 studies (n≈700); Observational cohorts	Serious¹	Presence of cognitive impairment (assessed using Mini-Cog, MMSE, CDT) was associated with increased risk of postoperative delirium and other complications. Meta-analysis not performed.	LOWMajor Limitations: Risk of bias: Observational studies with potential for confounding factors.Heterogeneity: Variability in the screening tools used (e.g., Mini-Cog, MMSE, CDT).Imprecision: Lack of pooled quantitative estimates.
Association of nutritional status with postoperative complications	5 studies (n≈650); Observational cohorts	Serious¹	Presence of malnutrition (assessed using MNA, MNA-SF) was associated with increased risk of complications and prolonged recovery. Meta-analysis not performed.	LOWMajor Limitations: Risk of bias: Moderate/High risk of bias in the primary studies.Inconsistency:Inconsistent results across studies.Indirectness: Not all studies were specific to dentistry.
Applicability and effectiveness of geriatric assessment tools in dental practice	12 studies (n=5651); Observational cohorts	Serious¹	A wide range of tools was identified (over 20 scales). No single validated protocol exists. Tools demonstrate feasibility for screening, but their impact on specific dental outcomes (e.g., implant failure) has not been studied.	VERY LOWVery serious limitations: Risk of bias: A high proportion of studies had a moderate/high risk of bias.Indirectness: The outcome was applicability, not efficacy.Heterogeneity: Extreme variability in tools and methodologies.

¹Serious risk of bias primarily due to confounding and missing data.²Risk of bias and methodological limitations in the included studies were considered when downgrading evidence quality according to GRADE.

## Data Availability

The datasets used and/or analyzed during the current study are available from the corresponding author.
